# Simultaneous fNIRS and thermal infrared imaging during cognitive task reveal autonomic correlates of prefrontal cortex activity

**DOI:** 10.1038/srep17471

**Published:** 2015-12-03

**Authors:** Paola Pinti, Daniela Cardone, Arcangelo Merla

**Affiliations:** 1 Infrared Imaging Lab – ITAB Institute for Advanced Biomedical Technologies and Department of Neuroscience, Imaging and Clinical Sciences, University of Chieti-Pescara, Italy

## Abstract

Functional Near Infrared-Spectroscopy (fNIRS) represents a powerful tool to non-invasively study task-evoked brain activity. fNIRS assessment of cortical activity may suffer for contamination by physiological noises of different origin (e.g. heart beat, respiration, blood pressure, skin blood flow), both task-evoked and spontaneous. Spontaneous changes occur at different time scales and, even if they are not directly elicited by tasks, their amplitude may result task-modulated. In this study, concentration changes of hemoglobin were recorded over the prefrontal cortex while simultaneously recording the facial temperature variations of the participants through functional infrared thermal (fIR) imaging. fIR imaging provides touch-less estimation of the thermal expression of peripheral autonomic. Wavelet analysis revealed task-modulation of the very low frequency (VLF) components of both fNIRS and fIR signals and strong coherence between them. Our results indicate that subjective cognitive and autonomic activities are intimately linked and that the VLF component of the fNIRS signal is affected by the autonomic activity elicited by the cognitive task. Moreover, we showed that task-modulated changes in vascular tone occur both at a superficial and at larger depth in the brain. Combined use of fNIRS and fIR imaging can effectively quantify the impact of VLF autonomic activity on the fNIRS signals.

Several studies demonstrated that the autonomic nervous system (ANS) is not just an “involuntary” and “non-cognitive” system (see Hugdahl^1^ for a review) as it is involved in any cognitive and emotional processes^2^,^3^. In fact, the afferent fibers of the ANS provide feedbacks to the brain thus letting the autonomic efferent control centers in the central nervous system (CNS) to modulate the autonomic outflow^1^.

Systemic changes are largely due to the homeostatic response of the human body when any mechanical or psychological stimulus, either external or internal, occurs. In particular, under mental stress, the main systems involved are the hypothalamic–pituitary–adrenal (HPA) system and the autonomic nervous system. Their activation brings to behavioral and peripheral changes for restoring the homeostatic balance. The ANS takes part in the homeostasis control, not only by regulating body functions (i.e., heart, gastro-intestines, glandular secretion, and so on), but also playing an important role in the processing of emotional experiences^2^,^4^.

In this study we combined simultaneous recording of cortical activity and autonomic arousal in order to study the autonomic and the neural processes underlying cognitive functioning^2^,^5^.

Brain activity was estimated by means of functional Near Infrared Spectroscopy (fNIRS). fNIRS is a non-invasive technique for optical estimation of cortical activity. It relies on the low optical absorption of the biological tissue of the infrared radiation in the 650–1000 nm wavelength window (the so-called optical window). fNIRS measures the relative changes in concentration of oxygenated (oxyHB) and deoxygenated (deoxyHB) hemoglobin, taking advantage of their absorption coefficients in the optical window^6^.

Since neural activity is accompanied by changes in blood oxygenation (i.e., increase of oxyHB and decrease of deoxyHB), fNIRS is particularly suitable to detect cortical hemoglobin correlates of functional cortical activation^7^,^8^,^9^. For a comparison between fNIRS and the other neuroimaging techniques, see Ferrari and Quaresima (2012)^6^ for a review.

However, fNIRS is limited by the penetration depth of the light and by the impossibility to provide anatomical information of the brain, thus limiting the investigation mostly to the cortical tissue^6^,^10^. Moreover, since fNIRS collects the backscattered light over the scalp, it is sensitive to the optical processes that happen at extra-cerebral layers of the head, as the light has to pass through them (then being also either partially absorbed and scattered). The recorded signal thus results in a mixture of stimulus-evoked and non-evoked components of both neural and systemic origin^11^. Over the past few years, researchers have focused their work on the potential effect of systemic task-evoked changes on the fNIRS estimation of cortical hemodynamics^12^,^13^,^14^,^15^,^16^,^17^,^18^. These studies suggest that systemic changes to cognitive task represent a source of noise over the detected brain activity signals and need to be reduced. In particular, Kirilina *et al.*^14^ assessed that task-evoked systemic artifacts are related to the sympathetic control of vessel walls, given the involvement of the autonomic activity in any cognitive and emotional process.

Spontaneous systemic changes (i.e. non-evoked vasomotion oscillations) need to be considered, too. They occur at different frequencies and include heartbeat (∼1 Hz), respiration (∼0.3 Hz), Mayer waves (∼0.1 Hz) and very low frequencies (VLF; <0.1 Hz)^11^. Although they are non-evoked by stimulation, their amplitude is task-modulated and may corrupt the optical estimation of the cortical response^8^,^11^.

In order to investigate the origin of the slower systemic changes, we estimated the autonomic responses to cognitive processes using functional thermal infrared (fIR) imaging^19^. fIR imaging is a non-contact and non-invasive technique that allows - by recording the heat radiated from the skin - to measure and to image the cutaneous temperature distribution and to estimate at a distance a series of autonomic parameters, among which the variation of the cutaneous perfusion^20^, the heart beat rate^21^, the sudomotor response^22^, up to emotional responses and arousal conditions through their thermal signature^19^,^23^,^24^,^25^,^26^. fIR imaging provides a powerful method to record the temperature changes and their physiological fluctuations, such as those related to sympathetic regulation of peripheral vascular tone^27^, since skin temperature is a function of superficial blood flow (SBF).

In fact, the ANS nerves are continuously active providing a certain degree of contraction and relaxation of the innervated blood vessels^27^, regulating SBF. Previous studies^28^ identified five frequency bands in which skin blood flow oscillations occur corresponding to the endothelium-related metabolic (0.008–0.02 Hz), neurogenic (0.02–0.05 Hz), myogenic (0.05–0.15 Hz), respiratory (0.15–0.4 Hz) and cardiac regulations (0.4–2 Hz).

Since fNIRS relies on neurovascular coupling and the vascular tone is regulated both by ANS muscles fibers and nerve plexuses^29^, we expected to find a relation between the thermally-recorded ANS responses and the frontal cortical activity by means of fNIRS during a mental arithmetic task^18^,^30^,^31^,^32^,^33^.

This task impacts on the cognitive workload as it involves the integration of complex mental processes, such as number recognition, mental calculation, decision making, responding^34^,^35^.

Prefrontal cortex (PFC) activity is related to working memory processes^34^ and it is activated during arithmetical tasks^5^,^31^,^36^,^37^. It is also easily reachable by NIR light. Moreover, previous studies demonstrated that PFC is involved in the modulation of autonomic responses and it plays a key role in the integration of the central and the autonomic nervous systems (see Van Eden and Buijs^35^ for a review).

NIRS measurements were taken using a multi-distance setup, collecting signals at different source-detector distances. The laser sources were placed at 4 cm, 3 cm, and 2 cm from the detector over the forehead of the participants. The longer distance channels (i.e., the deepest) provide information about the cortex activity^10^. The signals coming from the shorter channels (i.e., the more superficial) are more affected by systemic interferences due to forehead skin perfusion^13^,^14^,^17^,^38^.

Regarding thermal measurements, we focused on the temperature changes of the nose tip. This region is, in fact, strongly associated with the ANS activity^19^,^22^,^25^,^26^,^39^.

This study aims to evaluate the thermally-recorded autonomic correlates to cognitive functioning and to investigate the impact of the autonomic outflow on the optical estimation of brain activity. To the best of our knowledge, this is the first attempt to combine fIR imaging and fNIRS measurements for such investigation.

## Results

### fNIRS-fIR imaging experiment

#### fNIRS results

**Figure 1:**
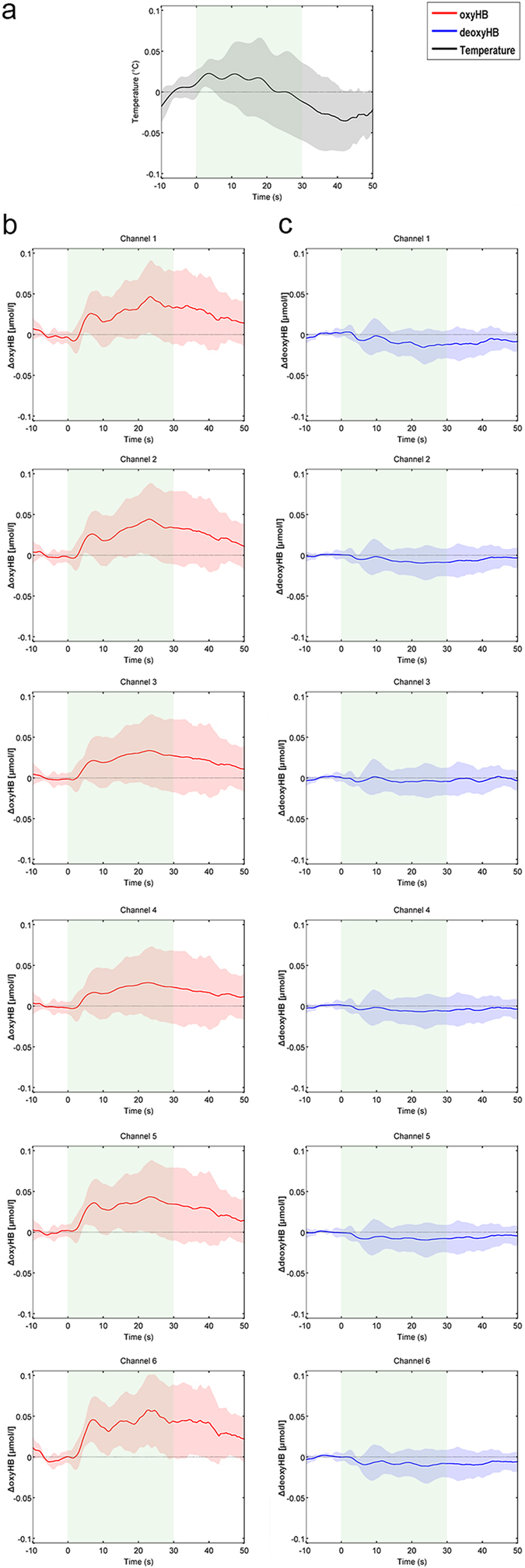
Grand average responses of nose tip temperature signal (a), oxyHB (b), and deoxyHB (c). The task period is indicated with green blocks. The colored shadowed areas represent the standard deviation of each curve.

Figure 1b,c show, for each of the six channels, the grand average responses for oxyHB and deoxyHB signals. Comparison of peak_oxyHB_ and peak_deoxyHB_ *vs*. 0-value have been performed to test the existence of a group activation. The results are showed in Table 1. We found a significant increase of oxyHB^35^,^40^ in all the channels. Significant decrements of deoxyHB were found for all the channels except for channel L3.

#### fIR imaging results

Task-evoked changes of temperature were observed for all the participants (Fig. 1a). The group mean temperature range is 0.32 ± 0.19 °C. The grand average signal is characterized by a periodicity lasting as long as the single block: the temperature rises during the rest phase reaching a plateau; during the task phase, it decreases with a delay of ∼10 seconds from the first stimulus; temperature returns to its initial level during the recovery phase.

#### Wavelet Analysis

**Figure 2:**
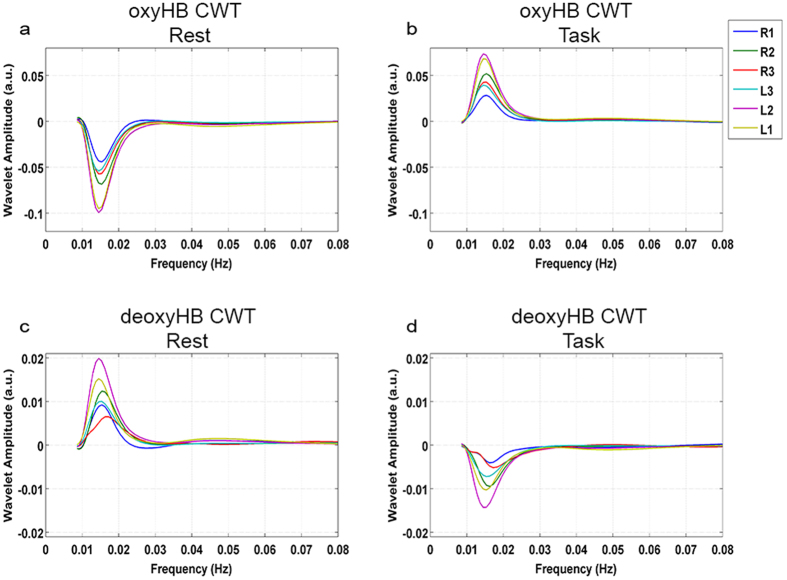
Grand average complex wavelet transform (CWT) for oxyHB during the rest (a) and the task (b) phase and for deoxyHB during the rest (c) and the task (d) phase, for all the channels.

**Figure 3:**
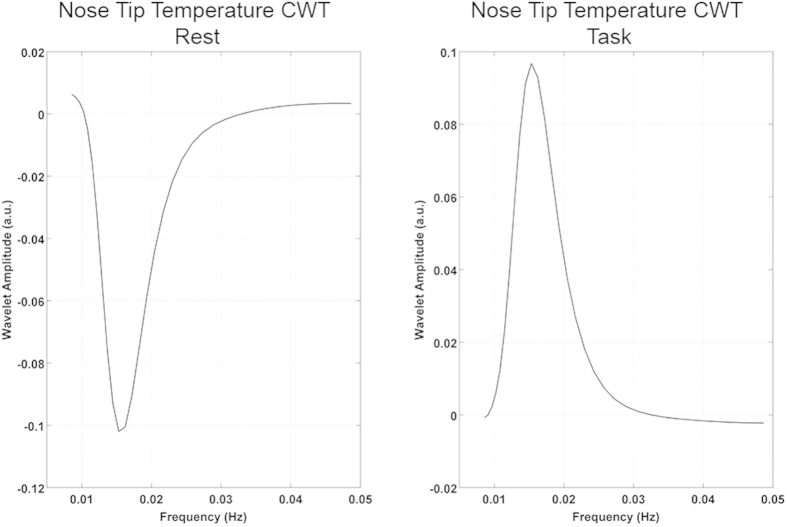
Grand average complex wavelet transform (CWT) for the nose tip temperature signal during the rest (left panel) and the task (right panel) phase.

Complex wavelet transforms of the fNIRS and fIR signals were computed to investigate the frequency content variations over time of each signal. Results are illustrated in Figs 2 and 3.

Wavelet coefficients corresponding to the grand average CWT of the oxyHB signals (Fig. 2a,b) were negative for the rest phase and positive for the task phase. Wavelet coefficients for the deoxyHB grand average CWT behaved oppositely (Fig. 2c,d). For all the channels, and for both oxyHB and deoxyHB, the maximum and the minimum values of the wavelet transform coefficients corresponded to ∼0.015 Hz. High positive and negative coefficients corresponded respectively to a strong correlation and anticorrelation between the mother wavelet and the signal at that scale.

The same result came out from the grand average spectra of nose tip temperature (Fig. 3). The sign inversion of the wavelet coefficients from the rest to the task phase suggests a task modulation of the VLFs both on the cortical and the autonomic signals.

**Figure 4:**
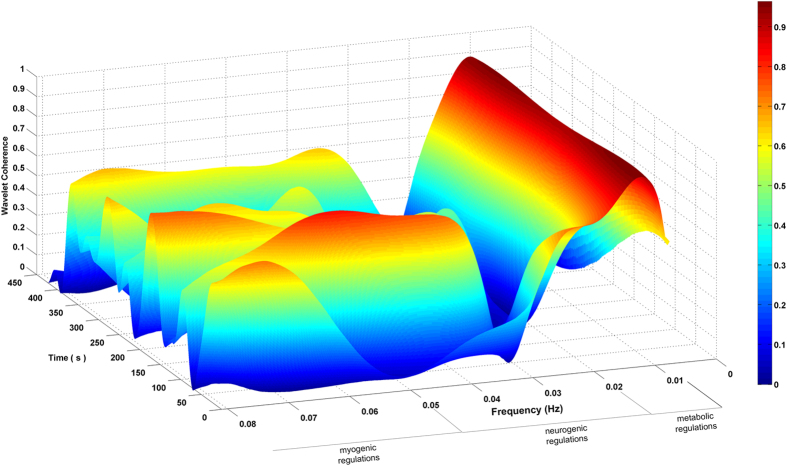
Wavelet Phase Coherence values (oxyHB-temperature) as a function of frequency and time of a representative participant.

In order to establish if the two signals are coherent, that is to establish if the VLF oscillations seen in fNIRS signals come from the autonomic skin vascular tone regulation, we calculated the Wavelet Phase Coherence values^41^ and the corresponding time lags between each of the six fNIRS channels’ signals and the nose tip temperature signal. Figure 4 shows the Wavelet Phase Coherence values (oxyHB-temperature) as a function of frequency and time of channel R1 for a randomly chosen participant. The higher coherence values did occur in the endothelium-related metabolic frequency band (f < 0.02 Hz).

In Table 2 the mean group value of Wavelet Phase Coherence during the task for each channel, both for oxyHB and deoxyHB is shown. We found that, for each channel, the temperature signal and the hemoglobin signals show almost the same power content in the VLFOs range. In particular, for both the hemispheres (channels R1-R2-R3 in the right hemisphere, channels L1-L2-L3 in the left hemisphere), the deeper channels (channel R1 and L1) show the higher coherence values within the same hemisphere group of channels.

These results suggest the existence of common components for the fNIRS and thermally-recorded ANS signals during cognitive functioning.

In Table 3 are listed the mean group time lags between each pair of signals. We tested the significance of mean group time lags through a one-sample t-test vs 0 (two-tailed, Bonferroni adjusted for multiple comparisons, p < 0.008). No significant time lags were found in all the channels and in all pairs of signals.

**Figure 5:**
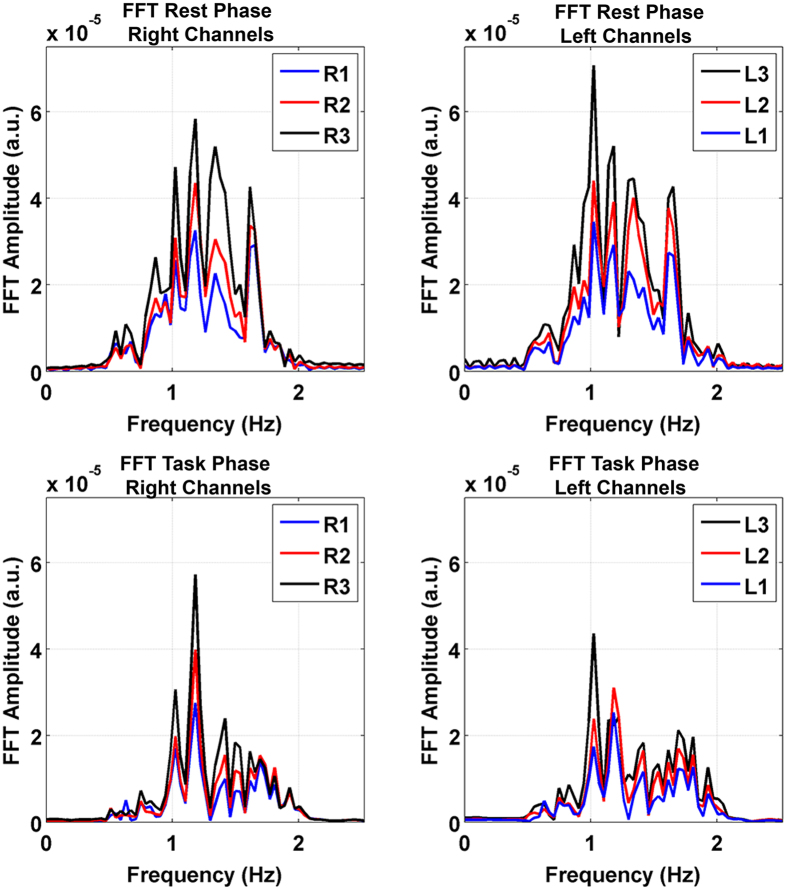
Grand average power spectra of the six NIRS channels during the rest (upper panels) and the task phase (lower panels) in the right (left panels) and left (right panels) hemisphere.

Regarding the heart rate, we compared the peak frequencies, detected on the averaged power spectra for each participant, of the rest and task phase through a paired sample t-test (two-tailed, Bonferroni corrected for multiple comparisons, p < 0.008). We did not find any significant differences in heart rate between the two phases. Figure 5 shows the grand average of the signals power spectra for the right and the left channels, separately, corresponding to the rest and task phases. It can be noted that the peak amplitude of the frequency is higher for the superficial channels (Channel R3-L3) and it decreases as the depths increase.

### Control experiment

#### fIR imaging results

The group mean temperature range is 0.26 ± 0.06 °C for the experimental condition and 0.22 ± 0.07 °C for the control condition. Group Pearson correlation coefficients computed between the nose tip temperature signals of the experimental and control conditions are reported in Table 4. A one-sample t-test was used to test whether the z-transformed PCCs are statistically significant in the control group. The correlation between the nose tip temperature signals for the control and experimental conditions resulted not significant (t = 0.83; p = 0.43).

## Discussion

In this study we investigated the contamination of the hemodynamic changes due to brain activity (assessed by means of fNIRS) by the hemodynamic changes related to the ANS activity (assessed by means of thermal IR imaging).

According to the literature, cerebral activity and autonomic systemic activity are related each other^2^,^3^. The latter should be properly considered in the estimation of brain oxygenation by means of fNIRS^13^,^14^,^17^,^18^,^38^. fNIRS signals reflect the hemodynamic changes happening both at the extra-cerebral and cerebral compartments. In fact, task-related autonomic activity brings to peripheral changes, which overlap with task frequencies thus contaminating the fNIRS signal^13^. In addition to task-evoked systemic interferences, spontaneous systemic activity needs to be considered, too^11^. In the study of Obrig *et al.*^8^, non-evoked systemic oscillations resulted not directly elicited by the stimulation but their amplitudes were task-modulated^8^.

In order to monitor and evaluate the ANS response to the assigned cognitive task, we recorded the variations of the nose tip temperature across the experimental phases. Thanks to the strong connection between skin temperature and the ANS, thermal imaging represents a powerful method to assess the peripheral systemic behavior^42^. The nose tip region was chosen for two main reasons: i) It reflects the sympathetic vasomotor activity^39^, thus depending on the cutaneous blood perfusion^19^,^25^; ii) it is not directly and anatomically related to the frontal and pre-frontal cortex, thus being able to unveil the autonomic connoted of the responses to the task. Cutaneous blood perfusion is known to be a potential source of artifacts for fNIRS^13^,^14^,^43^. Moreover, thermal cameras do not interfere with NIRS optodes like other instrument do (e.g. the light emitted by Laser Doppler^14^).

fNIRS measurements were taken over the prefrontal cortex since this region is involved in cognitive processing^34^. More precisely, we chose an arithmetical task. This task produced a significant increase of oxyHB in all the channels and a significant decrease of deoxyHB in all the channels except channel L3 (Table 1), in agreement with previous literature^5^,^31^,^40^. This may be due to the fact that deoxyHB changes have smaller amplitude than oxyHB variations thus resulting in a lack of statistical significance.

Concerning the temperature, we found a task-evoked ANS response (Fig. 1a). The superficial temperature decrement measured through fIR confirms the engagement of ANS during cognitive functioning^2^,^3^. The temperature decrease corresponds to a peripheral (i.e., cutaneous) vasoconstriction due to an increment of the sympathetic alpha-adrenergic vasoconstrictor effect. Vasoconstriction leads to a decrement of blood perfusion, which in turn determines a decrement of the skin temperature. We could therefore indirectly evaluate the sympathetic skin blood flow regulation during cognitive processing in a simple and objective way^44^. This result is in agreement with the results obtained by Kirilina *et al.*^13^. They used MR-angiography to register extracranial signals over the forehead during a continuous performance task and observed a deacrease of the forehead veins signal, phase locked to the stimulus onset. We also observed a decrease of the temperature signal but with an offset of ∼10 seconds. This may be due to a different and delayed sympathetic response to a different task or to the different considered region (nose tip vs forehead).

Since fNIRS measures cortical activity through the neurovascular coupling, it is sensitive to blood-related variations^6^. This interference may have the same dynamics of brain activity or it may be located in the same anatomical target, thus hiding or enhancing the fNIRS estimation of cortical activity.

Thermal and optical responses to task (Fig. 1) let us suppose the presence of a common physiological process underlying both the ANS and brain activity. Wavelet transform was thus used to evaluate the frequency content of fNIRS and fIR signals over time^41^,^45^. From the rest to the task phase, wavelet coefficients invert their sign both for oxyHB, deoxyHB and nose tip temperature. The transition from negative values during the rest to positive values during the task for the oxyHB (Fig. 2a,b) and temperature (Fig. 3) wavelet transform (and the opposite for the deoxyHB (Fig. 2c,d) indicates changes in a common physiological phenomena. This result suggests a task-modulation of the VLF components^8^.

A Wavelet Phase Coherence analysis was employed in order to evaluate the intensity of the covariance of the cortical and autonomic activities in a time-frequency space revealing regions with high common power^41^. Moreover, it unveils information about the phase differences between the two time-series. Wavelet Phase Coherence analysis over the fNIRS and the temperature signals (Table 2) proved a strong relation between each pair of cortical-autonomic signals, both for oxyHB and deoxyHB, in the VLFOs frequency band (0.008–0.08 Hz). In fact, blood supply to the skin requires the integration of different processes that work in parallel resulting in slow periodic fluctuations of temperature of the order of minutes^42^. Wavelet Phase Coherence coefficients resulted high also for the deoxyHB-temperature pairs suggesting that slow vasomotion changes occurs also in the venous compartment. In Fig. 4 we reported an example of Wavelet Phase Coherence in the time-frequency space for a randomly chosen participants. Cortical and autonomic signals show a common power content for f < 0.02 Hz in all the participants. This band corresponds to the metabolic endothelium-related regulation band. As this range doesn’t overlap with task frequencies, it is reasonable to suppose that these oscillations are not directly elicited by the task (i.e. spontaneous) but modulated by it^11^. Slow frequency oscillations in the fNIRS signals thus originate from the systemic endothelial regulation of the contractile state of vascular smooth muscles. Moreover, we can suppose that task-modulated vascular tone changes occur (Table 2) both superficially and more deeply in the brain, thus representing a source of interference on the optical estimation of cortical activity. In fact, the autonomic neural control is involved in the regulation of cerebral blood flow and both processes are related to the participant’s cognitive load^46^,^47^. Thus, fIR is suitable to measure and detect VLFOs linked to regulatory processes in the skin. It has been demonstrated that the autonomic responses represent a source of physiological noise in the fNIRS signals^12^,^13^,^14^,^17^,^48^. The temperature decrease reflects the superficial decrement of blood perfusion, and so of the amount of oxygenated hemoglobin. This likely may bring to an underestimation of the cortical activity recorded by fNIRS, as the NIR light absorption and back-scattering was not only influenced by the brain activity, but it was also less absorbed because of the decrement of blood volume.

Time lags (Table 3) between the task-evoked changes of the cerebral blood flow and of the superficial cutaneous blood flow show a high inter-participant variability. Also group time lags reported by Kirilina *et al.*^14^ in the VLFOs band have higher variability than the results obtained in the Mayer wave and respiration frequency ranges. The cognitive and/or the emotional state of the participant may thus modulate more the autonomic control of the vascular tone rather than the autonomic control of respiration, blood pressure or heart beat rate leading to higher inter-participants differences. In fact, thermal imprints are the best indicators of stress-induced mood changes^24^. Our results (Table 3) suggest that inter-participant variability in the VLFOs band needs to be considered when fNIRS signal are de-noised through a GLM approach^14^. Physiological regressors in the VLFOs range may be constructed on the base of the single-participant’s cognitive/emotional state as it impacts on the phase differences between autonomic and cerebral responses.

We did not find significant changes in the cardiac frequency, as estimated through the NIRS signal, among experimental phases. This may be due to a different autonomic control of the task-related superficial vasodilatation mechanism with respect to the autonomic innervation of the heart^18^. The peak amplitudes of the frequency spectra depend on the penetration depth of the light. As shown in Fig. 5, channel R3 and L3 have the highest peak amplitude and it decreases when the depth increases. This finding indicates that the physiological noise generated by heartbeat, as well as the sympathetic interference, seems to have a stronger effect on the superficial channels^13^,^38^.

In order to demonstrate that the facial temperature modulations found in our study are task-related and are not just spontaneous oscillations, we have included a control experiment. Nose tip temperature changes corresponding to experimental condition did not significantly correlate with the nose tip temperature changes corresponding to control condition (Table 4). This result demonstrates that the temperature changes found in the present study are evoked responses reflecting the overall autonomic nervous system response to the cognitive task.

Even though the present study has to be considered preliminary, it introduces some relevant findings. The cognitive-emotional link and the role that one system exerts on the other could be effectively studied by simultaneously combining thermal IR and fNIRS imaging, while preserving the ecological context of the participant’s natural actions. It could be thought that such link could be studied even at a single-participant level. In addition, thermal IR does not interfere with NIRS system, as it is touch-less and passive. The influence of the familiarity of the task on the sympathetic response is also avoided since the measurements are taken simultaneously. Moreover, the use of additional instruments, such as thermal cameras, is useful in case of a NIRS system with a limited number of optodes. In fact, superficial signal regression techniques^12^,^38^,^48^ require a larger number of optodes. To achieve a good signal improvement, each NIRS channel must be combined with a short separation channel as close as 1.5 cm^38^.

As a side result, this study also suggests to consider that thermal autonomic effects are appreciable when executing a cognitive task. However, temperature fluctuations are slow processes and thus might not follow cognitive tasks at a faster pace. Cortical hemodynamic processes are slow as well thus making fNIRS and fIR well suited to be combined. In fact, it is interesting to note that the temperature response exhibit a similar pattern of the hemodynamic response (Fig. 1a). This likely proves the involvement of the ANS on cognitive functioning. These implications need to be considered when correcting for short channel separations as sympathetic interference distribution may vary over the forehead and occurs not only superficially^18^,^38^,^48^. Moreover, the results of our study suggest to use a participant-based fNIRS signal de-noising as the cognitive and/or the emotional state of the participant modulates the interference timing.

However, this study presents several limitations. The first one is the small sample size. It is needed to enlarge it, even though the statistical evidences we found are pretty strong. It would be interesting to enrich the study with the concurrent recording of a wider set of autonomic processes (e.g., ECG, blood pressure, skin conductance) and to evaluate how the autonomic effects vary with different block designs and tasks. Another limitation is that we did not separate the spontaneous systemic interference from the fNIRS signal. Future works are needed to combine fIR-fNIRS measures over the forehead to evaluate the task-related autonomic interference directly on the region of interest.

## Materials and Methods

### Participants

Eighteen healthy participants took part in this study (mean age: 26.6 ± 3.7 years; 6M/12F). Nine participants performed the combined fNIRS-fIR experiment and the other nine were enrolled for a control experiment and underwent only fIR measurements. However, the fNIRS probe was placed on their forehead to exactly replicate the same experimental conditions of the combined fNIRS/thermal experiment. Two samples of participants were included in this study in order to avoid habituation effects or loose of interest to the assigned cognitive task.

Given the potential influence on the fIR imaging signals, information about the recreational drug use and medical and psychological history were assessed in a telephone interview. Regular recreational drug users (cannabis within the last two months, other recreational drugs within the past year), habitual smokers (>7 cigarettes/week) and individuals reporting chronic illness (e.g., cardiovascular or thyroid conditions), psychological disorders (e.g. depression or anxiety) or taking medication that influences hypothalamic-pituitary-adrenal regulation were excluded from the study^24^. The study was approved by the Research Ethics Board of the University of Chieti-Pescara and performed in agreement with the Declaration of Helsinki. All participants signed informed consent and could withdraw from the study at any time.

### Procedure

Prior to the experiment, participants acclimated for about 15 minutes within the experimental room, which was kept at steady temperature and humidity (23 ± 1 °C; 50–60% relative humidity), and without any direct air ventilation on the participants.

Participants were asked to avoid smoke, alcoholic and caffeinated beverages for at least 2 hours prior the experimental session in order to minimize the vasoactive effects that these substances have on the skin temperature.

Stimuli were presented on a 19-inch computer screen by using a custom software, implemented in Matlab (The MathWorks Inc., Natick, Massachusetts).

## fNIRS-fIR experiment

The experimental paradigm included 6 activation blocks. Each block included: a 15-seconds of pre-stimulus period (rest); a task phase, lasting maximum 32 seconds; and a post-stimulus recovery phase, lasting 15 seconds. This phase was excluded from further analyses as it was included in the experimental protocol to let the variables (temperature and hemoglobin concentrations) to return to their baseline values after the stimulation. The task consisted of responding to 3 consecutive arithmetic subtractions (5 digits minus 3 digits), where a constant subtrahend was subtracted to the result of the previous instance, (e.g. 17235–271 = 16964, 16964–271 = 16693, 16693–271). Each subtraction was presented for 5 seconds, and the participants were asked to choose the proper answer from a list of 4 possible results. A maximum of 7-seconds time period was allowed for providing the correct answer to each subtraction. If a wrong answer occurred within a block, the participants had to restart the computation from the first subtraction of that block. The responses were collected using a computer keyboard. The number of wrong/exact answers as well the time required for providing each answer were recorded.

All the subtraction operations were randomized among participants and blocks.

## Control experiment

A control experiment was performed as well in order to assess if facial temperature modulations are the expression of the functional response to the cognitive task and are not spontaneous oscillations overlapping the evoked response just by chance. The control experiment thus included two conditions, in a counterbalanced order across participants:

- an experimental condition, in which participants performed the same cognitive task of the fNIRS-fIR experiment;
- a control condition, in which participants looked at a fixation cross for the same duration of the experimental condition, without performing any task.

### Functional Near-Infrared Spectroscopy Measurement

**Figure 6:**
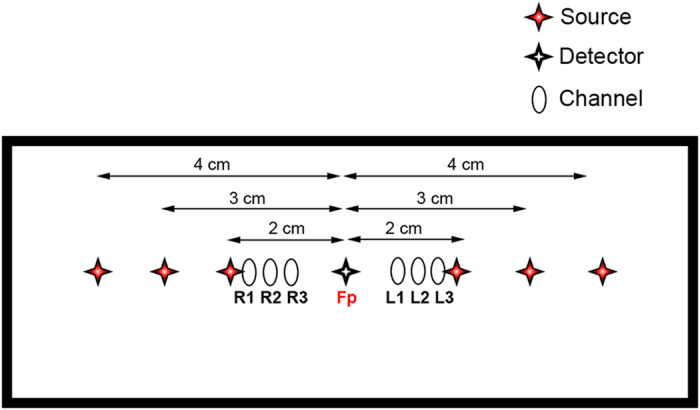
NIRS sources (S) and detector (D) arrangement and channels configuration.

In order to investigate the prefrontal cortex activity, we used a commercial frequency-domain near-infrared spectroscopy instrument (Imagent, ISS Inc., Champaign, IL). The instrument provides 32 laser diodes sources - 16 emitting light at 690 nm and 16 at 830 nm - modulated at 110 MHz and 4 photomultiplier-tubes (PMT) modulated at 110.005 MHz. The sources were time-multiplexed during the data acquisition (switch mode: 16; sampling frequency: 10 Hz). The light power emitted by the diodes at the fiber end was <4 mW∕cm^2^, within the ANSI standard limits and thus permitting safe measurements. We used 6 sources for each wavelength and one detector, resulting in 6 measurements channels, and we plugged them into a multi-distance pad configuration. The PMT was placed at the center of the pad and the sources were placed symmetrically to the right and the left of the PMT at 2 cm, 3 cm, and 4 cm from the PMT in order to evaluate the NIRS signal at different depths (Fig. 6). The channels in the right and left hemispheres are indicated with R1-R2-R3 and L1-L2-L3, respectively, ordered by decreasing depth.

Since the prefrontal cortex is involved in higher cognitive functions^5^,^40^,^49^, the holder was placed over the forehead of the participant according to the international 10–20 electrode placement system, on the Fp1-Fp2 line with the PMT detector in correspondence of the Fp point^50^.

### Processing and Statistical Analysis of fNIRS data

The pre-processing of fNIRS data started with the assessment of the quality of the signals in order to discard those channels with a low Signal-to-Noise ratio (SNR) due to a poor scalp-optode coupling (e.g., because of the hair)^10^. As the holder was placed over the forehead, the scalp coupling was good enough to avoid discarding any channels among all of the participants. fNIRS data have been pre-processed using the Homer2 NIRS Processing package^51^.

The intensity signals were first converted into optical density (OD) signals for each source-detector pair. Motion artifacts were identified as signals variations larger than 5% of the standard deviation of the signal within a time-period of 1 second. A channel-based cubic spline interpolation (p = 0.99) was used to correct for motion errors^52^. OD signals were then band-pass filtered (0.007–0.5 Hz), in order to reduce slow drifts and physiological noises (e.g., heart rate), and converted into variation in concentration of oxygenated and deoxygenated hemoglobin using the modified Beer-Lambert law^53^. The quality of the signals has been improved by using the correlation-based signal improvement (CBSI) correction^52^. Cortical signals were down sampled to 2 Hz and a block-average was then applied to obtain the mean Hemodynamic Response Function (HRF) for each channel of each participant. No trials have been discarded in all the participants. The mean HRFs have been corrected with respect to the baseline by subtracting the mean value of each rest period to the corresponding task block. Also, for each channel, the grand average of each hemodynamic signal has been computed, as well (Fig. 1b,c).

For all the participants and for each channel, the absolute peaks of oxyHB (peak_oxyHB_) and deoxyHB (peak_deoxyHB_) were obtained from the participant’s mean HRF in a time window from 2 s to 25 s after the stimulus onset. This window was chosen to include the maximum variations of oxyHB and deoxyHB. To assess statistically significant activations, one-sample t-tests (two-tailed) were performed to compare the participants’ peak_oxyHB_ and peak_deoxyHB_ against zero. To control for false positives errors associated with multiple testings, we used the Bonferroni correction for all our statistical tests. The significance level α = 0.05 was thus divided by the number of comparisons (e.g. the number of channels) resulting in an adjusted significance level of 0.008.

In order to verify if any changes in the heart beat rate occurred across the experimental phases, we extracted the heart rate from the total hemoglobin signal. For each participant and for each channel, the unfiltered OD data have been band-pass filtered between 0.5 Hz and 2.5 Hz, since this range includes the major part of the cardiac oscillations. After converting OD data into variation of concentrations, we computed the Fast Fourier Transform of the rest and task phase on the total hemoglobin signal. We then averaged the power spectra across the blocks. We computed the heart rate through the peak frequency. A paired sample t-test (two-tailed, Bonferroni adjusted for multiple comparisons, p < 0.008) evaluated whether statistically significant heart beat changes occurred across the phases. All the statistical analyses were carried on by using the SPSS software (SPSS Inc., Chicago, Illinois, U.S.A.).

### Functional Thermal Infrared Measurements

**Figure 7:**
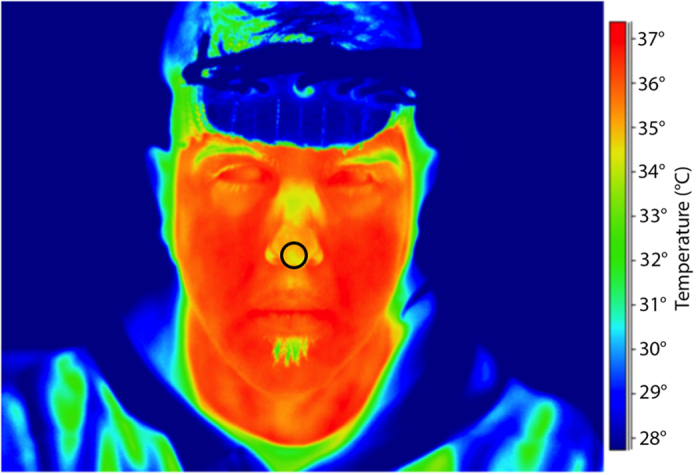
Facial thermogram of a representative participant. The black circle indicates the selected nose tip region.

To record the facial temperature changes for the entire duration of the experiment, we used a digital infrared camera (FLIR SC660, 640 × 480 bolometer FPA; thermal sensitivity/Noise Equivalent Temperature Difference (NETD): <30 mK @ 30 °C, FOV: 24° × 18°), placed 60 cm far away from the participant and pointed toward the face of the participant (Fig. 7). The sampling frequency was set at 2 Hz. In order to null noise effects related to potential drift/shift of the sensor’s response and optical artifacts, the camera response was blackbody-calibrated.

### Thermal data processing and statistical analysis

For all the participants and for both the fNIRS-fIR and the control experiment, the time series of the facial thermal images were first visually evaluated to assess the quality of the recordings. After having selected the nose tip as region of interest, the time course of its temperature was extracted. Since the participants were allowed to move the head without any restriction, a soft-tissue tracking algorithm was used to track the nose tip across all of the images of the time-series, in order to properly compute the nose tip temperature from each facial thermogram. The tracking software was developed with an in-home developed Matlab-based algorithm validated in Manini *et al.*^25^. It is based on the 2-D cross-correlation between a template region, chosen by the user on the first frame, and a similar ROI in a wider searching region, expected to contain the desired template in each of the following frames.

The thermal signal was further corrected from residual motion artifacts. Motion errors were visually detected and corrected by substituting them with the mean value of the 6 samples before and after the artifact. Then we applied a Butterworth band-pass filter (0.007–0.15 Hz) to remove physiological noises (e.g., thermal effects associated with respiration) and slow drifts. Temperature range has then been computed on the preprocessed thermal signals of the fNIRS-fIR experiment and the control and experimental condition of the control experiment for each participant and then averaged across the corresponding sample to get a group mean temperature range.

For the fNIRS-fIR experiment, the temperature time course has then been averaged over each block for each participant to obtain a mean temperature response to the task. The mean temperature signal has been corrected with respect to the baseline by subtracting the mean value of the rest period from each task block. The grand average of all thermal data has been computed, as well (Fig. 1a).

For the control experiment, Pearson correlation coefficients (PCC) were computed between the nose tip temperature signals of the experimental and control conditions for each control participant. This was done to assess if the temperature modulations during the experimental condition are task-related and are thus poorly correlated to the control condition temperature oscillations. A Fisher r-to-z transform was applied to the PCC of each participant reported in Table 4. The z-values were tested against zero using a one-sample t-test in order to assess if significant correlations occur between the experimental and the control condition.

### Wavelet Analysis

In order to investigate the link between the cortical activity (measured through the fNIRS signal) and the autonomic nervous system response (measured through the thermal signal) during the cognitive task in the fNIRS-fIR experiment, wavelet transform was applied to both the fNIRS and thermal time series. Wavelet analysis was performed through the Matlab package provided by Grinsted *et al.*^41^ (http://noc.ac.uk/using-science/crosswavelet-wavelet-coherence). The wavelet transform provides a multiresolution analysis decomposing the signals into a time-frequency space. The signal power content can be thus evaluated as a function of frequency and time^41^. The complex wavelet transform (CWT) of the cerebral and autonomic signals was computed using a Morlet mother wavelet, scaled and translated over time^14^,^54^. CWT was applied on the band-pass filtered data in order to focus on the physiological frequency range of interest (0.008–0.08 Hz). The frequencies centered at 0.1 Hz (known as Mayer waves) were also excluded as they are related to oscillations in arterial pressure rather than local vasomotion^55^. Equidistant scales ranging from 12.5 seconds to 100 seconds (spaced of 0.2 seconds) were hence chosen as vasomotion regulations occur in the VLFOs range 0.008–0.08 Hz^11^,^27^,^55^ and the most of the energy of temperature oscillations are found at low frequencies. The complex wavelet transform was calculated for each of the fNIRS signals (both for oxyHB and deoxyHB) recorded by the six channels and the temperature signal. To evaluate the effect of cognitive processes on the VLFOs, the wavelet transforms were averaged within each time scale in each block, for both task and rest, respectively. The mean wavelet transforms of the task and rest phase were then averaged across the six repetitions. The grand average wavelet transform across all the participants were computed as well.

In order to quantify the intensity and the phase differences between the cerebral and the autonomic activity, we applied a Wavelet Phase Coherence Analysis^14^,^28^,^54^,^56^,^57^. Wavelet Phase Coherence (WPC) evaluates the correlation of two non-stationary signals as a function of time and frequency^41^,^45^. The amplitude of the WPC indicates the intensity of the covariation between two time-series and its phase reveals information about the phase differences (i.e. time delay) between the two signals^41^. We thus calculated WPC^41^ between each of the fNIRS channels signals (both oxyHB and deoxyHB) and the nose tip temperature signal.

For each participant and for each pair of signals, a mean WPC magnitude in each task block was calculated within each time scale. Mean WPC magnitude was then averaged across the blocks. The same procedure was applied to the phase of WPC using the circular mean instead of the arithmetical mean to take into account the circular nature of angular data. The maximum value of coherence and the corresponding phase difference (converted into time lags) were collected from the averaged coherence and phase signals.

Statistical significance of time lags was assessed contrasting the group time lags against zero through a one-sample t-test (two-tailed, Bonferroni adjusted for multiple comparisons, p < 0.008).

## Additional Information

**How to cite this article**: Pinti, P. *et al.* Simultaneous fNIRS and thermal infrared imaging during cognitive task reveal autonomic correlates of prefrontal cortex activity. *Sci. Rep.* **5**, 17471; doi: 10.1038/srep17471 (2015).

## Figures and Tables

**Table 1:** Result of the contrast of peak_oxyHB_ and peak_oxyHB_ *vs*. 0-value (Bonferroni corrected for multiple comparisons, p < 0.008).

Cerebral activation results				
Channels	oxyHB		deoxyHB	
	t	p	t	p
R1	4.6	0.0017	−4.9	0.0013
R2	4.8	0.0013	−4.9	0.0011
R3	5.4	0.0006	−5.6	0.0006
L3	3.6	0.0067	−3.1*	0.0157
L2	3.6	0.0077	−3.9	0.0046
L1	3.5	0.0079	−4.8	0.0014

Asterisks mark channels that didn’t survive the Bonferroni corretion.

**Table 2:** Mean group Wavelet Phase Coherence values and standard deviations computed between each of the six oxyHb and deoxyHB signals and the nose tip temperature signal during task execution.

Mean ± s.d. group Wavelet Phase Coherence values						
	R1	R2	R3	L3	L2	L1
oxyHB-Temperature	(0.75 ± 0.13)	(0.71 ± 0.17)	(0.66 ± 0.16)	(0.64 ± 0.18)	(0.63 ± 0.19)	(0.66 ± 0.14)
deoxyHB - Temperature	(0.75 ± 0.13)	(0.71 ± 0.17)	(0.66 ± 0.16)	(0.64 ± 0.18)	(0.61 ± 0.18)	(0.66 ± 0.14)

**Table 3:** Mean group time lags and standard deviations computed between each of the six oxyHb and deoxyHB signals and the nose tip temperature during task execution.

Mean ± s.d. group time lags (seconds)						
	R1	R2	R3	L3	L2	L1
oxyHB-Temperature	(−2.1 ± 15.2)	(−1.8 ± 14.3)	(2.4 ± 14.7)	(3.6 ± 14.2)	(1.1 ± 14.0)	(1.5 ± 11.7)
deoxyHB - Temperature	(4.1 ± 7.7)	(0.5 ± 9.3)	(4.2 ± 8.1)	(1.6 ± 9.5)	(4.0 ± 8.0)	(3.0 ± 10.2)

**Table 4:** Pearson correlation coefficients computed for each participant (P1–P9) between the nose tip temperature signals corresponding to the experimental and control condition.

Pearson Correlation Coefficients									
	P1	P2	P3	P4	P5	P6	P7	P8	P9
PCC	−0.10	0.15	−0.15	0.16	0.12	0.15	0.04	0.01	−0.08
